# Supplementary material for: Identification of a pathway for electron uptake in Shewanella oneidensis
Source: Commun Biol. 2021 Aug 11;4:957. doi: 10.1038/s42003-021-02454-x (PMC8357807; doi:10.1038/s42003-021-02454-x)
Supplement: Supplementary file 2 — Supplementary Information [file 42003_2021_2454_MOESM2_ESM.pdf]

Supplementary Information For:

Identification of a Pathway for Electron Uptake in *Shewanella oneidensis*

*Annette Rowe[*1†], Farshid Salimijazi[*2], Leah Trutschel[*1], Joshua Sackett[1], O. Adesina[3], I. Anzai[3], L. Kugelmass[3], M. Baym[4], Buz Barstow[2†]*

[*]denotes equal contribution to authorship

[1]Department of Biological Sciences, University of Cincinnati, Cincinnati, OH 45221, USA
[2]Department of Biological and Environmental Engineering, Cornell University, Ithaca, NY 14853, USA
[3]Department of Chemistry, Princeton University, Princeton, NJ 08544, USA
[4]Department of Biomedical Informatics, Harvard Medical School, Boston, MA 02115, USA

[†]Corresponding authors:

Annette Rowe, 731F Rieveschel Hall, University of Cincinnati, Cincinnati, OH 45221; annette.rowe@uc.edu

Buz Barstow, 228 Riley-Robb Hall, Cornell University, Ithaca, NY 14853; bmb35@cornell.edu

Supplementary Information Figures

Figure S1. Time course of $AHDS_{red}$ oxidation by wild-type *S. oneidensis*.

Figure S2. Representative time courses of anticipated hits from $AHDS_{red}$ oxidation screen of the *S. oneidensis* whole genome knockout collection.

Figure S3. Representative time courses of unanticipated hits from $AHDS_{red}$ oxidation screen of the *S. oneidensis* whole genome knockout collection.

Figure S4. Example electrochemical measurements of an *S. oneidensis* biofilm.

Figure S5. $AHDS_{red}$ oxidation rates and biological cathodic currents produced by selected mutants of *S. oneidensis*.

Figure S6. Aerobic to anaerobic and anaerobic to aerobic transitional growth curves of wild-type *S. oneidensis* mutants and selected mutants.

Figure S7. Comparison of biofilm morphology assessed by fluorescence microscopy between wild-type and *S. oneidensis* mutant SO_0841.

Figure S8. Phylogenetic tree constructed for 120-200 of the closest identified genes to *SO_0841* in the Integrated Microbial Genes Database.

Figure S9. Phylogenetic tree constructed for 120-200 of the closest identified genes to *SO_0181* in the Integrated Microbial Genes Database.

Figure S10. Phylogenetic tree constructed for 120-200 of the closest identified genes to *SO_0400* in the Integrated Microbial Genes Database.

Figure S11. Phylogenetic tree constructed for 120-200 of the closest identified genes to *SO_3660* in the Integrated Microbial Genes Database.

Figure S12. Phylogenetic tree constructed for 120-200 of the closest identified genes to *SO_3662* in the Integrated Microbial Genes Database.

Figure S13. Construction and usage of first generation miniature macroscope.

Figure S14. Construction and use of the large format macroscope.

Figure S15. Construction and use of second generation miniature macroscope.

Figure S16. Sample of images from the macroscope, part one.

Figure S17. Sample of images from the macroscope, part two.

Supplementary Information Tables

Table S1. Electrochemical data observed on cathodes for selected *S. oneidensis* transposon insertion mutants and controls.

Table S2. Growth and electrochemical data for deletion mutants of *SO_0181*, *SO_0400*, *SO_0841*, *SO_3660*, *SO_3662* and their corresponding complementation strains.

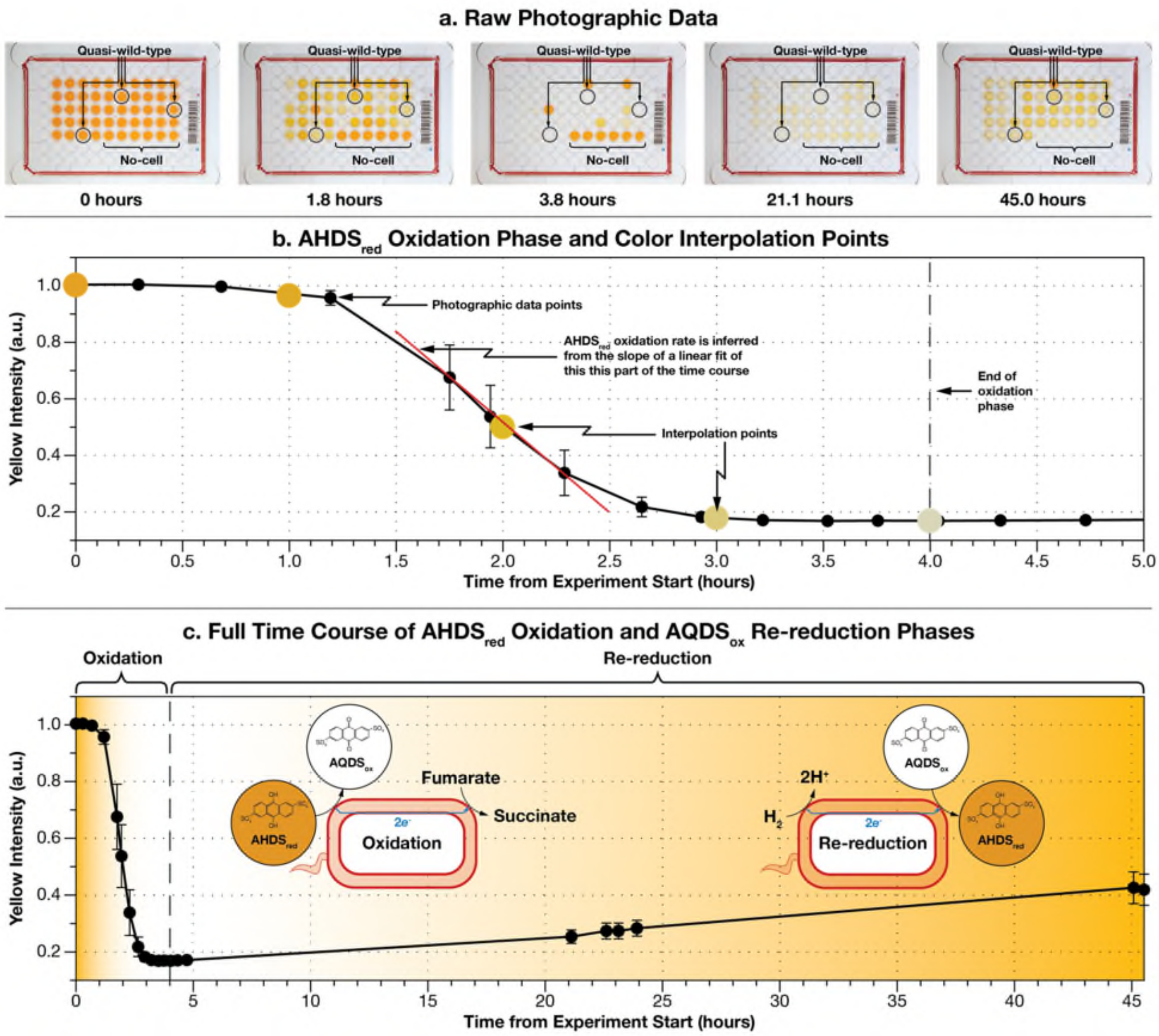

Figure S1. Time course of AHDS$_{red}$ oxidation by wild-type *S. oneidensis*. Quasi-wild-type mutants contain a transposon insertion but have no effect on AHDS$_{red}$ oxidation. (**a**) Raw photographic time-series of a single AHDS$_{red}$ oxidation assay plate. Mutants that behave like wild-type (quasi-wild-type) and no-cell controls are highlighted in the time-series of photographs. All wells in columns 1 and 12, and rows A, G and H are blank (no cells, and no AHDS$_{red}$/AQDS$_{ox}$). (**b**) Close up of the AHDS$_{red}$ oxidation phase (hours 0 to 4 from the start of the experiment) showing yellow intensity interpolation points used in the color graphs in **Figure 1** in the main text and linear fit used to calculate AHDS$_{red}$ oxidation rates reported in **Figure S5**. (**c**) Long time course of yellow intensity of average of quasi-wild-type wells showing AHDS$_{red}$ oxidation phase, and the subsequent re-reduction phase caused by transfer of electrons from H_2 in the headspace of the anaerobic chamber to AQDS$_{ox}$ mediated by *S. oneidensis*. Note that the no-cell controls slowly oxidize over ≈ 40 hours due to residual O_2 in the anaerobic chamber (< 20 ppm). Data is available in reference [1].

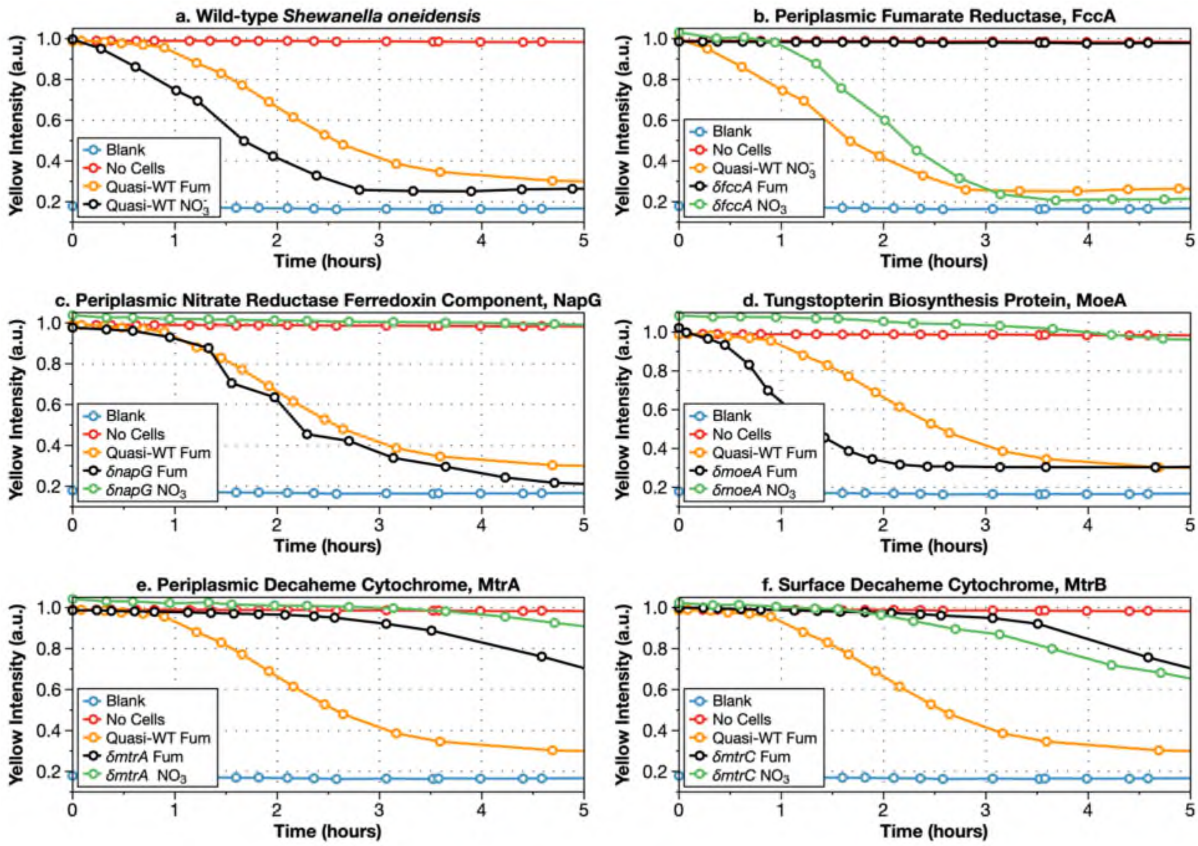

Figure S2. The $AHDS_{red}$ oxidation screen finds anticipated hits from the *S. oneidensis* whole genome knockout collection. (**a**) Quasi-wild-type *S. oneidensis* oxidizing $AHDS_{red}$ with fumarate (Fum) and nitrate (NO_3^-) terminal electron acceptors. (**b**) Disrupting the periplasmic fumarate reductase FccA knocks out $AHDS_{red}$ oxidation when fumarate is used as a terminal electron acceptor, but not nitrate. (**c** and **d**) Conversely, disruption of the nitrate reductase ferredoxin component (encoded by *napG*) or the MoeA enzyme that enables synthesis of its co-factor (coded by *moeA*) disrupts $AHDS_{red}$ oxidation when using nitrate as a terminal electron acceptor, but not fumarate. (**e** and **f**) Disrupting the MtrA and MtrC multi-heme cytochrome components of the Mtr EET complex slows $AHDS_{red}$ oxidation with both nitrate and fumarate. Data is available in reference [1].

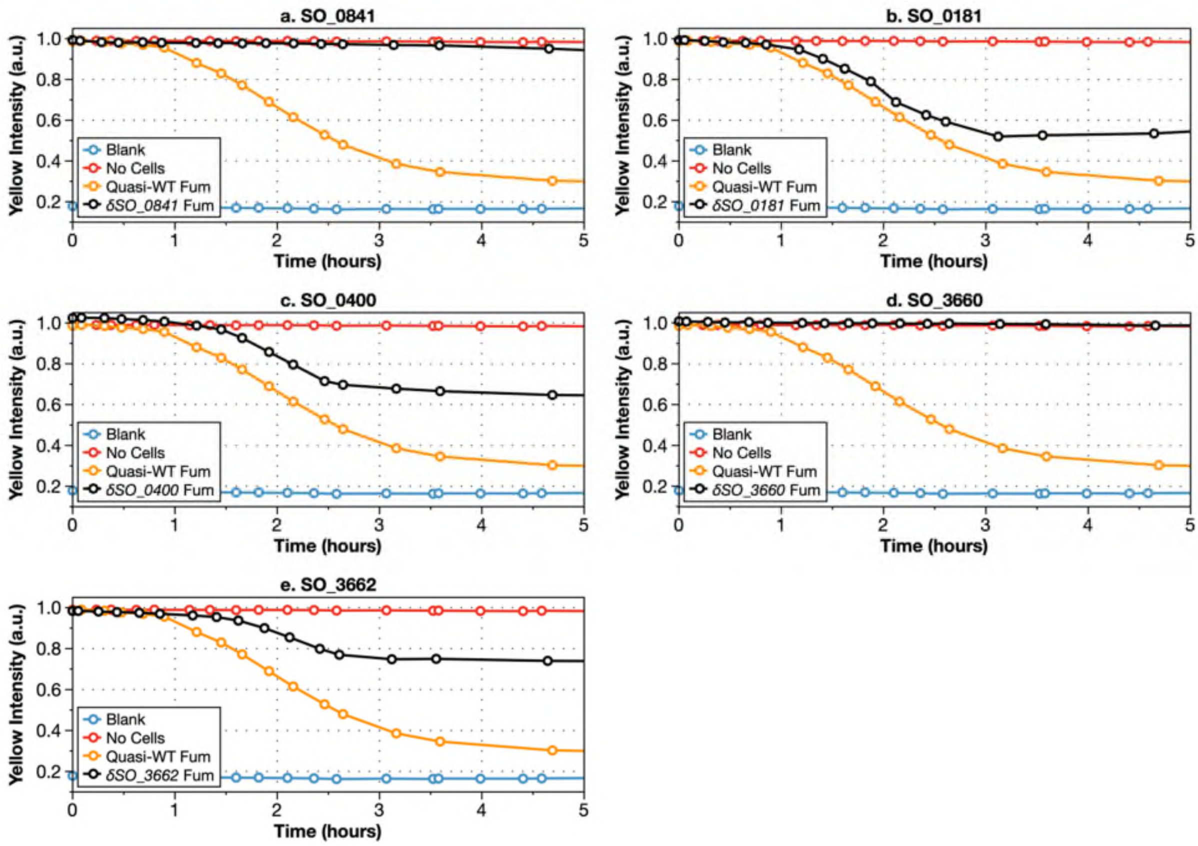

Figure S3. The $AHDS_{red}$ oxidation screen of the *S. oneidensis* whole genome knockout collection found 5 hits that produce robust disruption of electron uptake from a cathode. Here were show representative time courses of $AHDS_{red}$ oxidation coupled to fumarate reduction for disruption mutants of these 5 genes. Data is available in reference [1].

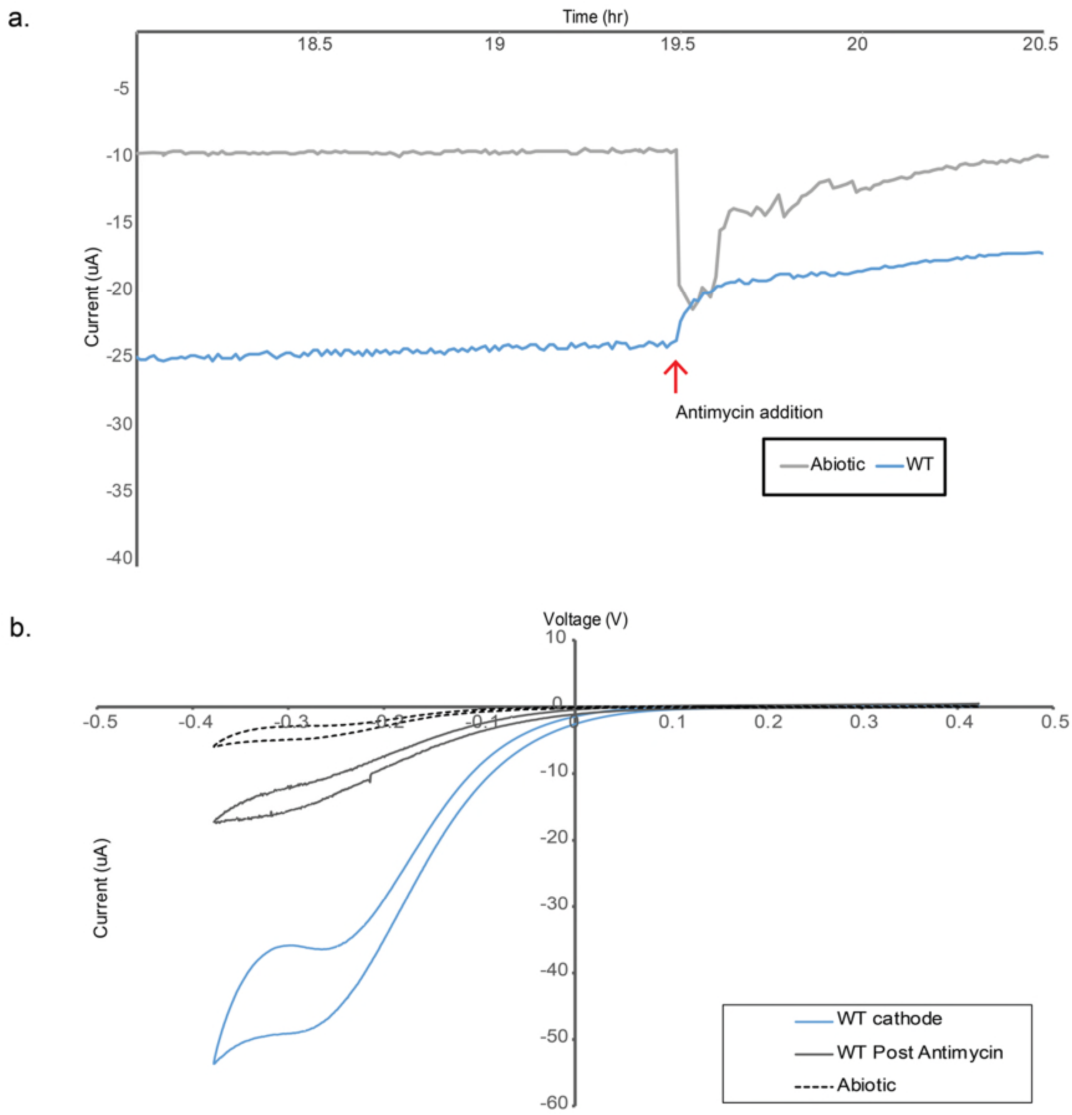

Figure S4. Example electrochemical measurements of an *S. oneidensis* biofilm. (**a**) Representative biological current production by wild-type *Shewanella oneidensis* MR-1 indicating total cathodic current and drop in current after the addition of the biological inhibitor Antimycin (50 µM) as compared with an abiotic control (n = 1). (**b**) Cyclic voltammograms of the corresponding abiotic, cathodic biofilm and Antimycin inhibited conditions for experiment shown in panel **a** (n = 1). Data is available in reference [1].

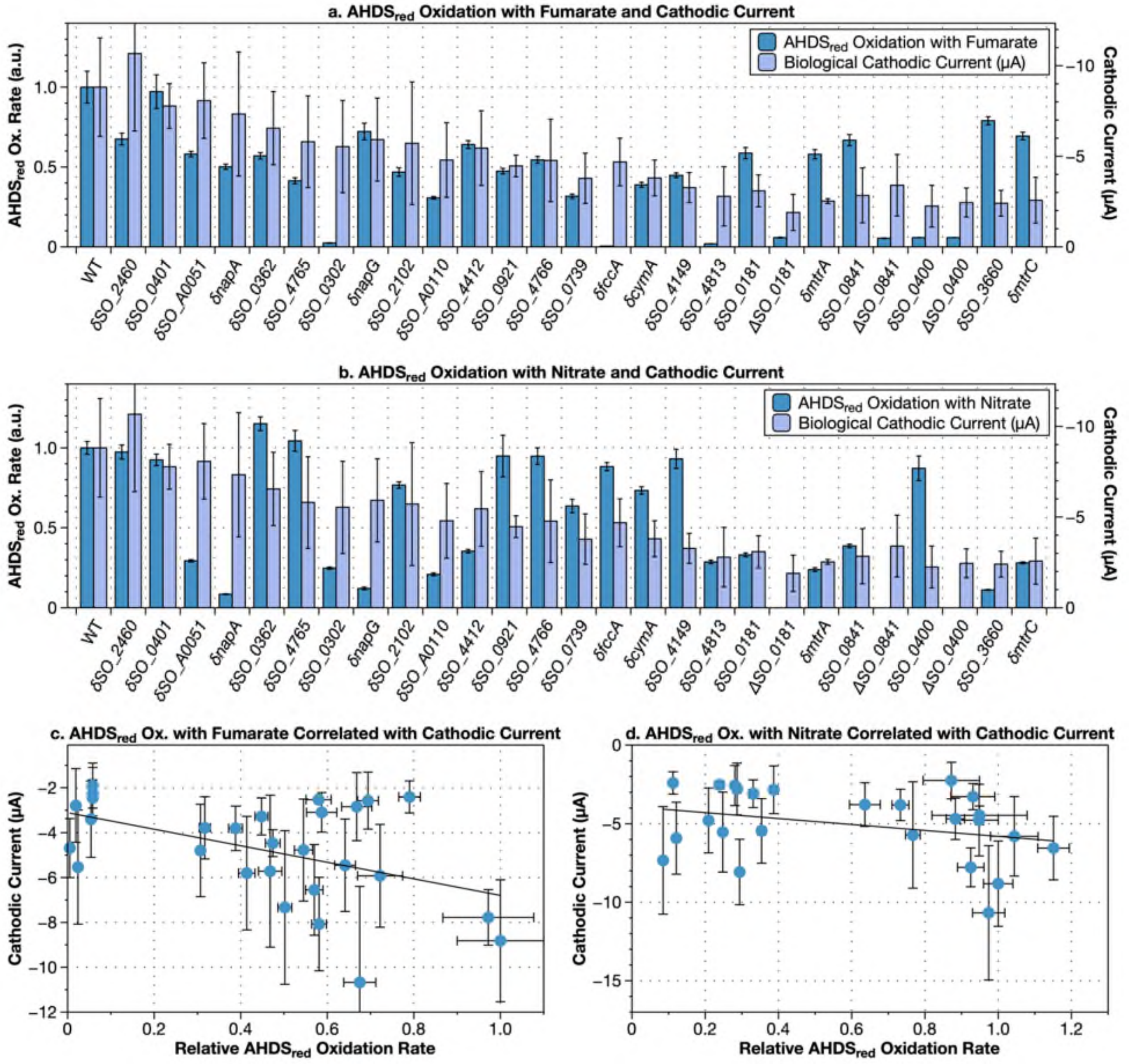

Figure S5. AHDS$_{red}$ oxidation rates and biological cathodic currents produced by selected mutants of *S. oneidensis*. Selected mutants are controls or produced AHDS$_{red}$ oxidation failure for unknown reasons. δ indicates a transposon insertion mutant, while Δ indicates a gene deletion mutant. The AHDS$_{red}$ oxidation rate is measured from the linear section of the yellow intensity trace as shown in **Figure S1** and is reported relative to the wild-type oxidation rate (for oxidation rates, $n = 3$). (**A**) AHDS$_{red}$ oxidation with fumarate and cathodic current. (**B**) AHDS$_{red}$ oxidation with nitrate and cathodic current. (**C**) AHDS$_{red}$ oxidation with fumarate correlated with cathodic current. (**D**) AHDS$_{red}$ oxidation with nitrate correlated with cathodic current. Data is available in reference [1].

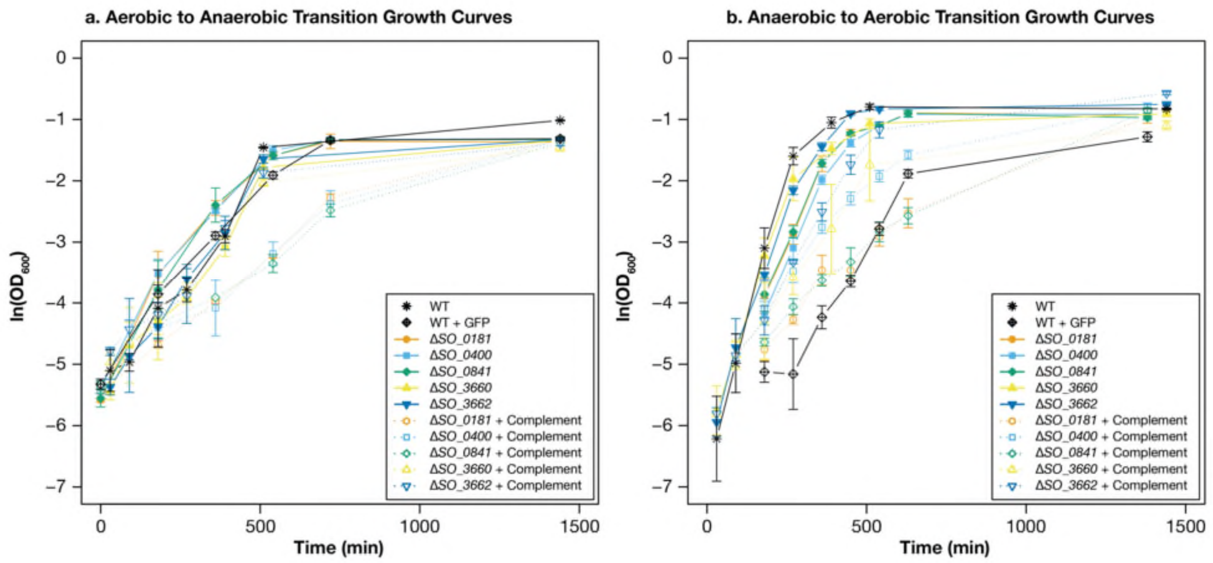

Figure S6. Aerobic to anaerobic and anaerobic to aerobic transitional growth curves of wild-type *S. oneidensis* mutants and selected mutants. (**A**) Anaerobic growth curves for cells pre-grown aerobically are shown for clean deletion mutants and complements of genes *SO_0181*, *SO_0400*, *SO_0841*, *SO_3660*, and *SO_3662*, compared to wild-type. (**B**) Aerobic growth curves of cultures pre-grown anaerobically for mutants and wild-type as listed above. Growth curves were measured in minimal media with 10 mM lactate as an electron donor and either oxygen or fumarate (20 mM) as an electron acceptor. Averages of triplicate batch cultures (n = 3) for each strain are illustrated with error bars indicating the standard deviations. Data is available in reference [1].

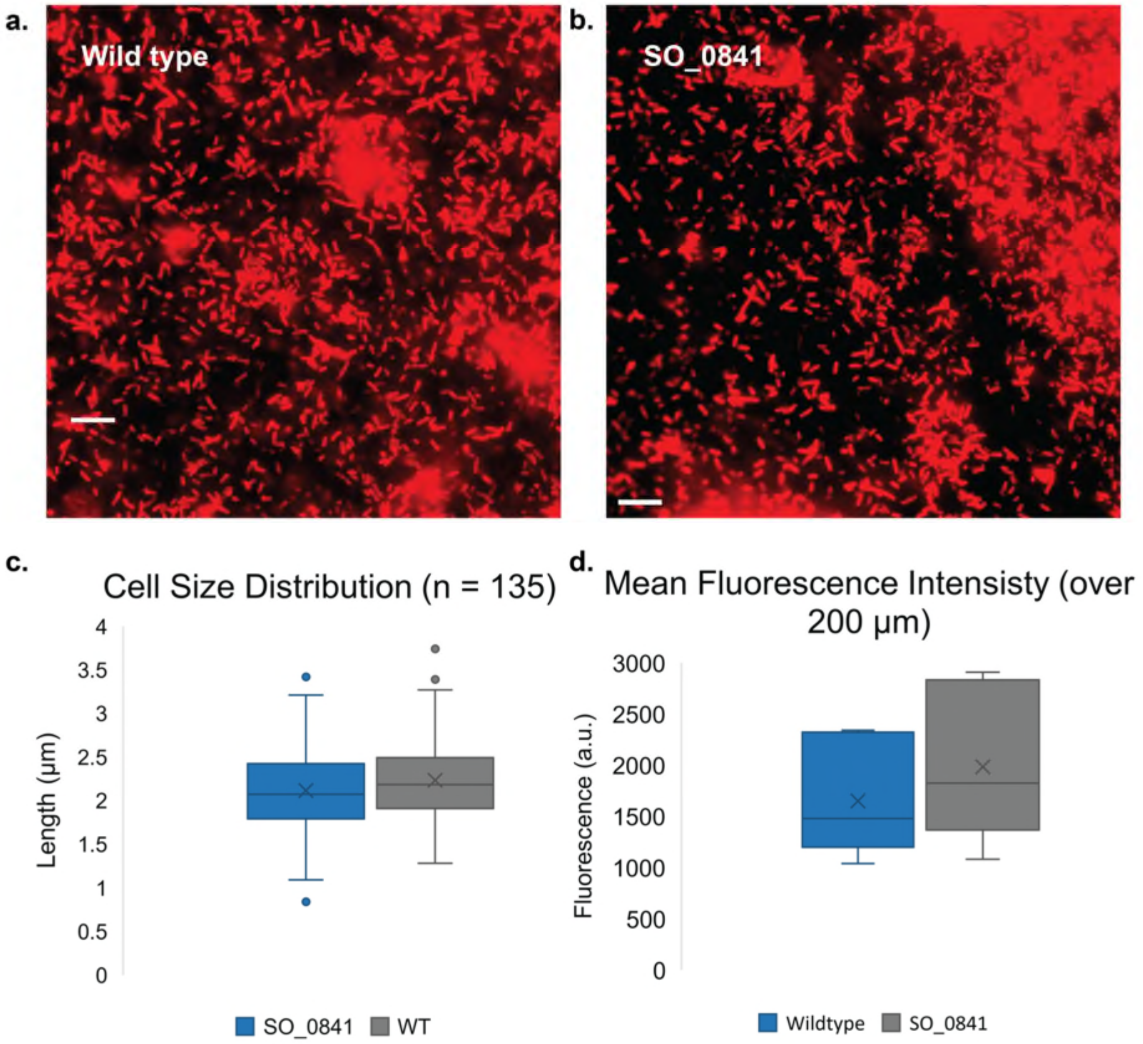

Figure S7. Comparison of wild-type and mutant biofilm morphology assessed by fluorescence microscopy. (**a**) Wild-type *S. oneidensis*. (**b**) *δSO_0841* gene disruption mutant. In panels **a** and **b** the cells were stained with a lipid stain. (**c**) No difference was observed between cell sizes measured (*n* = 135 cells from each condition measured) (**d**), and/or the mean fluorescence intensity over a 200 µm cross section section (*n* = 6 biologically independent reactors). Data is available in reference [1].

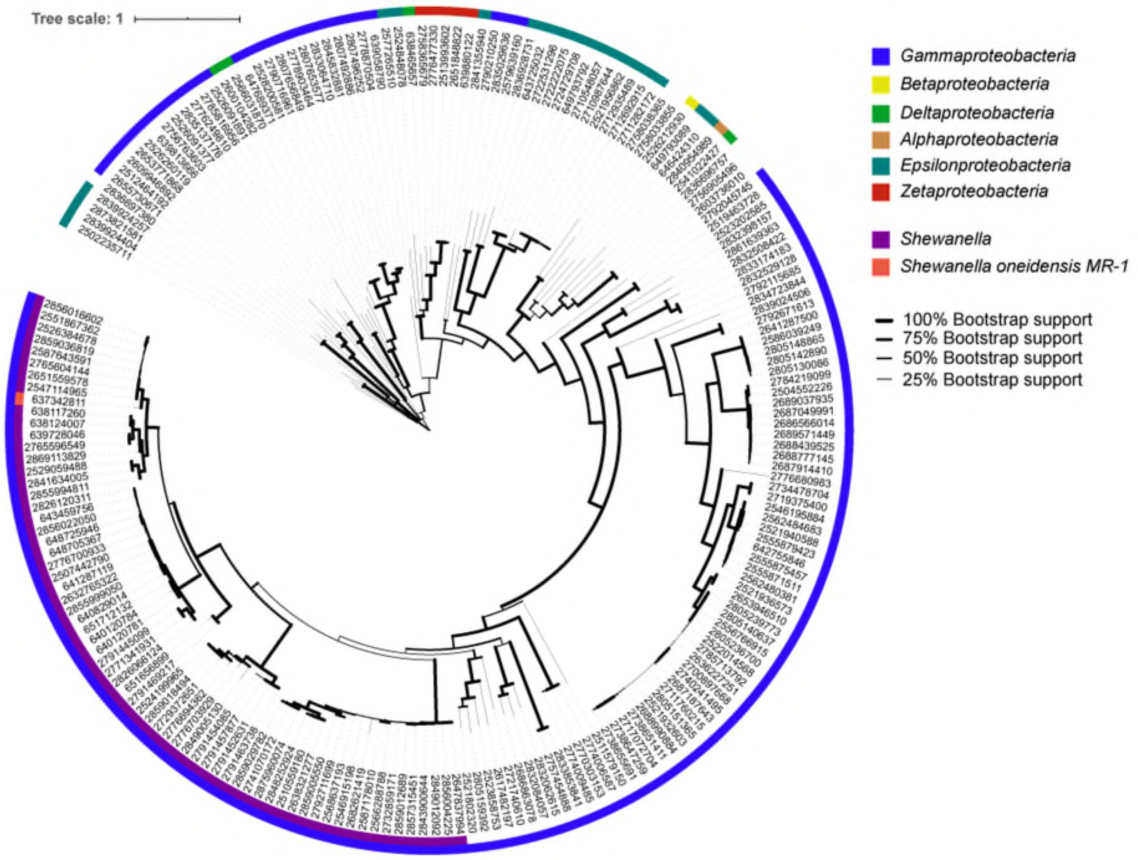

Figure S8. Phylogenetic tree constructed for 120-200 of the closest identified genes to *SO_0841* in the Integrated Microbial Genes Database (https://img.jgi.doe.gov/). Alignments generated using Muscle 3.8.425 using default parameters. A best scoring maximum likelihood tree was generated using RAxML 8.2.11 using 100 bootstrap replicates to identify the optimal tree. The tree was annotated using the interactive tree of life interface (https://itol.embl.de/). Thickness of branches indicates boot strap support for each branch. Color of outer-ring indicates phylum with a focus on *Proteobacteria*. Inner ring denotes homologs from *Shewanella* species with the strain identified in this study highlighted (*Shewanella oneidensis* MR-1). Metadata for trees attached in supplementary **Supplementary Data 2**.

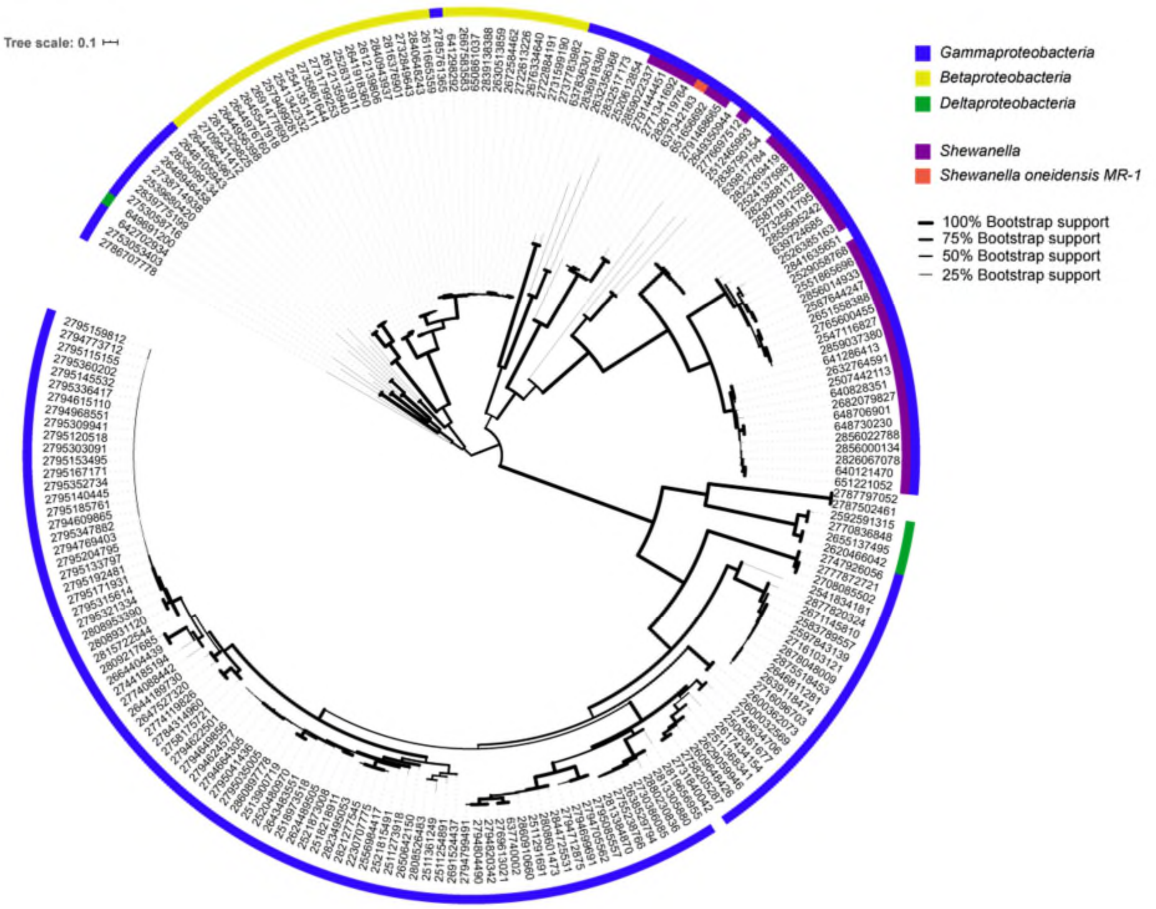

Figure S9. Phylogenetic tree constructed for 120-200 of the closest identified genes to *SO_0181* in the Integrated Microbial Genes Database (https://img.jgi.doe.gov/). Alignments generated using Muscle 3.8.425 using default parameters. A best scoring maximum likelihood tree was generated using RAxML 8.2.11 using 100 bootstrap replicates to identify the optimal tree. The tree was annotated using the interactive tree of life interface (https://itol.embl.de/). Thickness of branches indicates boot strap support for each branch. Color of outer-ring indicates phylum with a focus on *Proteobacteria*. Inner ring denotes homologs from *Shewanella* species with the strain identified in this study highlighted (*Shewanella oneidensis* MR-1). Metadata for trees attached in supplementary **Supplementary Data 2**.

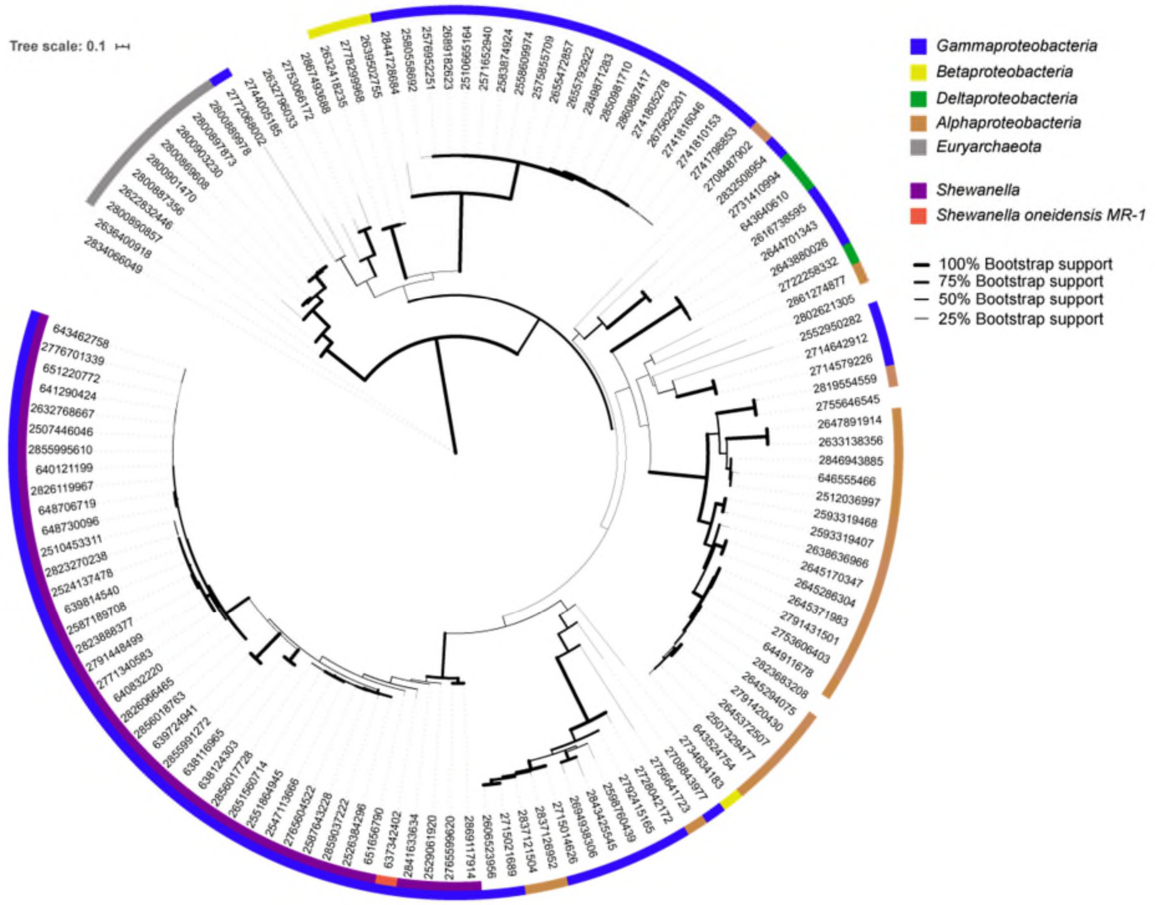

Figure S10. Phylogenetic tree constructed for 120-200 of the closest identified genes to *SO_0400* in the Integrated Microbial Genes Database (https://img.jgi.doe.gov/). Alignments generated using Muscle 3.8.425 using default parameters. A best scoring maximum likelihood tree was generated using RAxML 8.2.11 using 100 bootstrap replicates to identify the optimal tree. The tree was annotated using the interactive tree of life interface (https://itol.embl.de/). Thickness of branches indicates boot strap support for each branch. Color of outer-ring indicates phylum with a focus on *Proteobacteria*. Inner ring denotes homologs from *Shewanella* species with the strain identified in this study highlighted (*Shewanella oneidensis* MR-1). Metadata for trees attached in supplementary **Supplementary Data 2**.

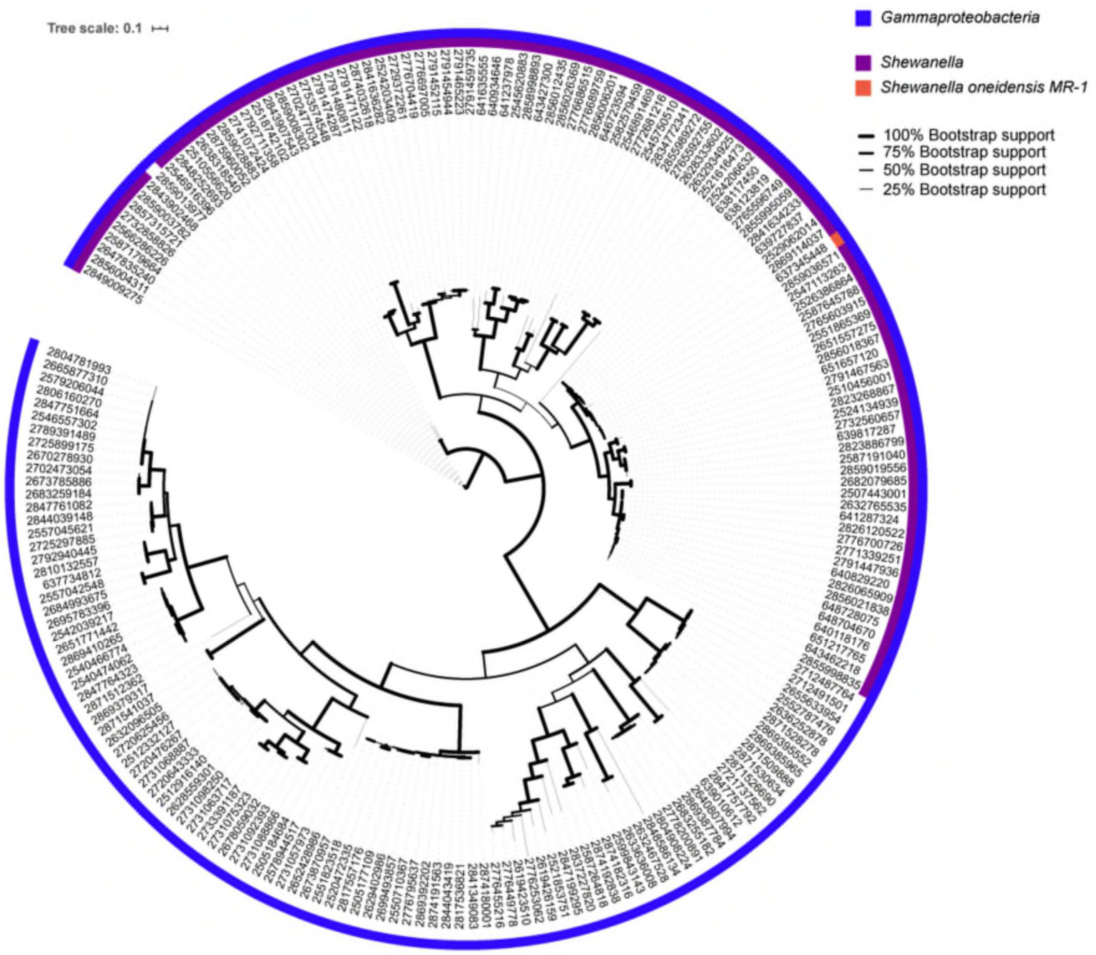

Figure S11. Phylogenetic tree constructed for 120-200 of the closest identified genes to *SO_3660* in the Integrated Microbial Genes Database (https://img.jgi.doe.gov/). Alignments generated using Muscle 3.8.425 using default parameters. A best scoring maximum likelihood tree was generated using RAxML 8.2.11 using 100 bootstrap replicates to identify the optimal tree. The tree was annotated using the interactive tree of life interface (https://itol.embl.de/). Thickness of branches indicates boot strap support for each branch. Color of outer-ring indicates phylum with a focus on *Proteobacteria*. Inner ring denotes homologs from *Shewanella* species with the strain identified in this study highlighted (*Shewanella oneidensis* MR-1). Metadata for trees attached in supplementary **Supplementary Data 2**.

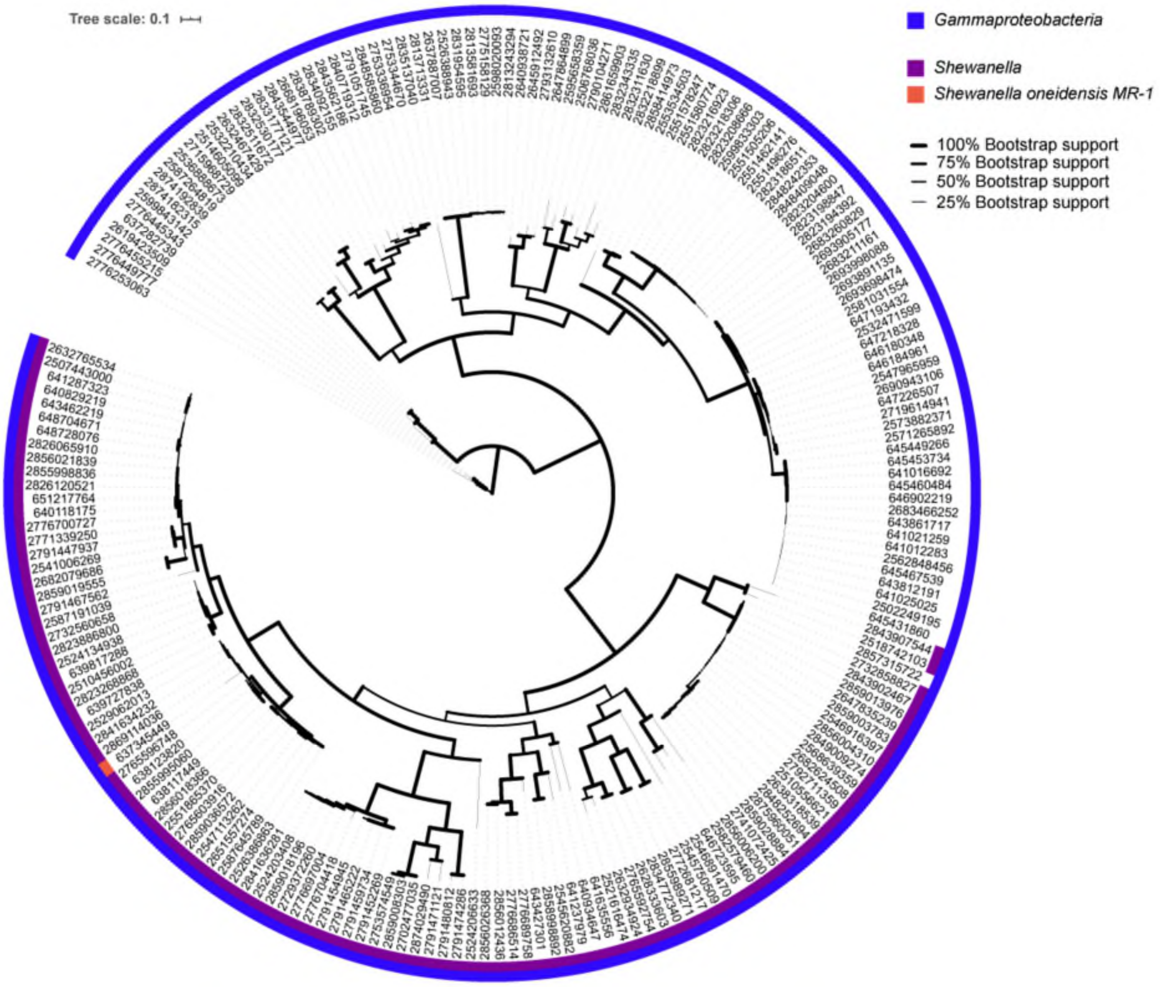

Figure S12. Phylogenetic tree constructed for 120-200 of the closest identified genes to *SO_3662* in the Integrated Microbial Genes Database (https://img.jgi.doe.gov/). Alignments generated using Muscle 3.8.425 using default parameters. A best scoring maximum likelihood tree was generated using RAxML 8.2.11 using 100 bootstrap replicates to identify the optimal tree. The tree was annotated using the interactive tree of life interface (https://itol.embl.de/). Thickness of branches indicates boot strap support for each branch. Color of outer-ring indicates phylum with a focus on *Proteobacteria*. Inner ring denotes homologs from *Shewanella* species with the strain identified in this study highlighted (*Shewanella oneidensis* MR-1). Metadata for trees attached in supplementary **Supplementary Data 2**.

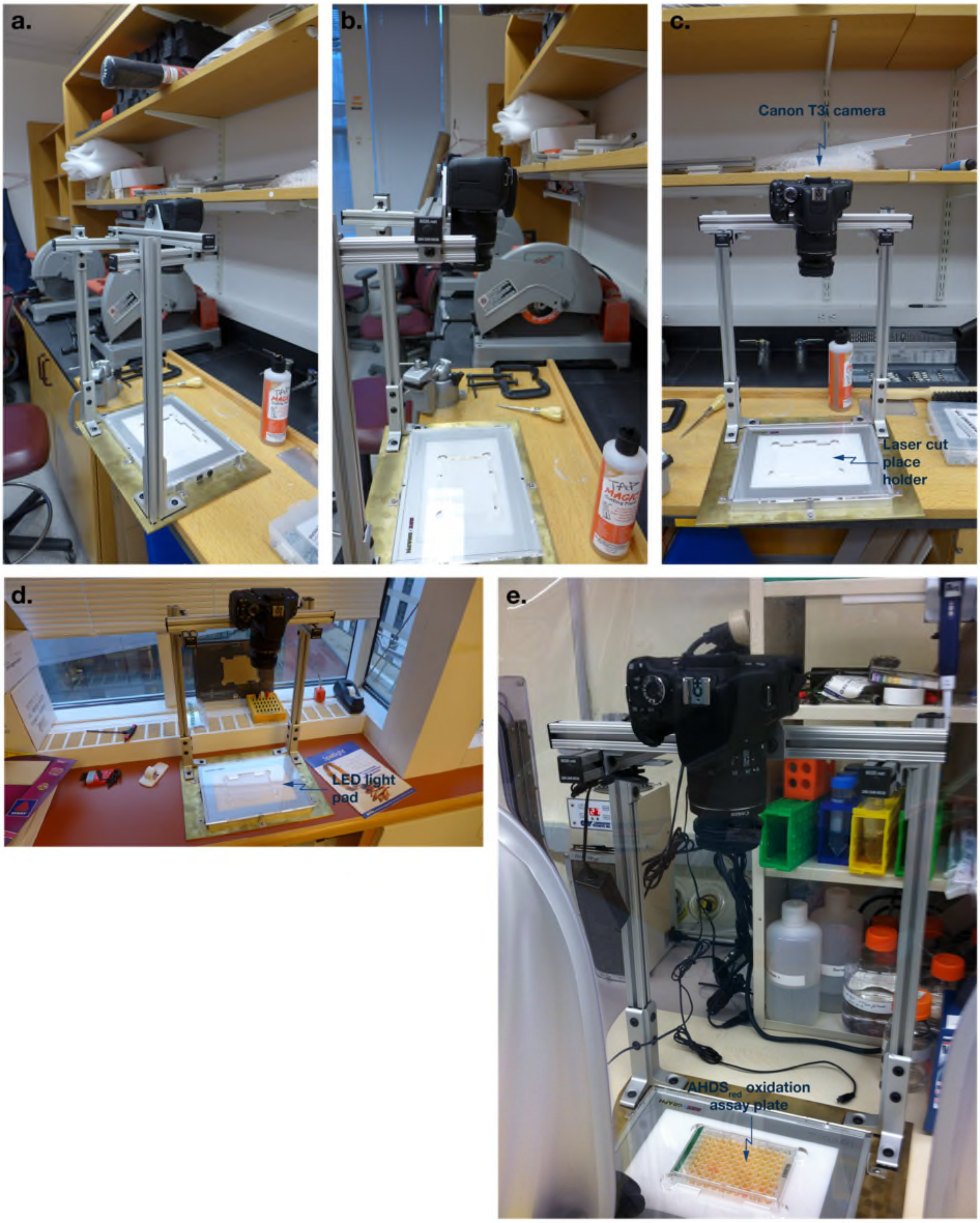

Figure S13. Construction and use of the first generation miniature macroscope. The miniature macroscope was used to collect photographs of assay plates inside a vinyl bag anaerobic chamber (Coy Laboratory Products) (**a** to **d**) The macroscope base was milled from scrap brass. The camera support frame was constructed from 1” extruded aluminum (T-slot). A place holder for SBS format micro-well plates was laser cut from acrylic and placed on top of an LED light pad (Artograph) (**e**) The miniature macroscope in use inside an anaerobic chamber ready to photograph an $AHDS_{red}$ oxidation assay plate.

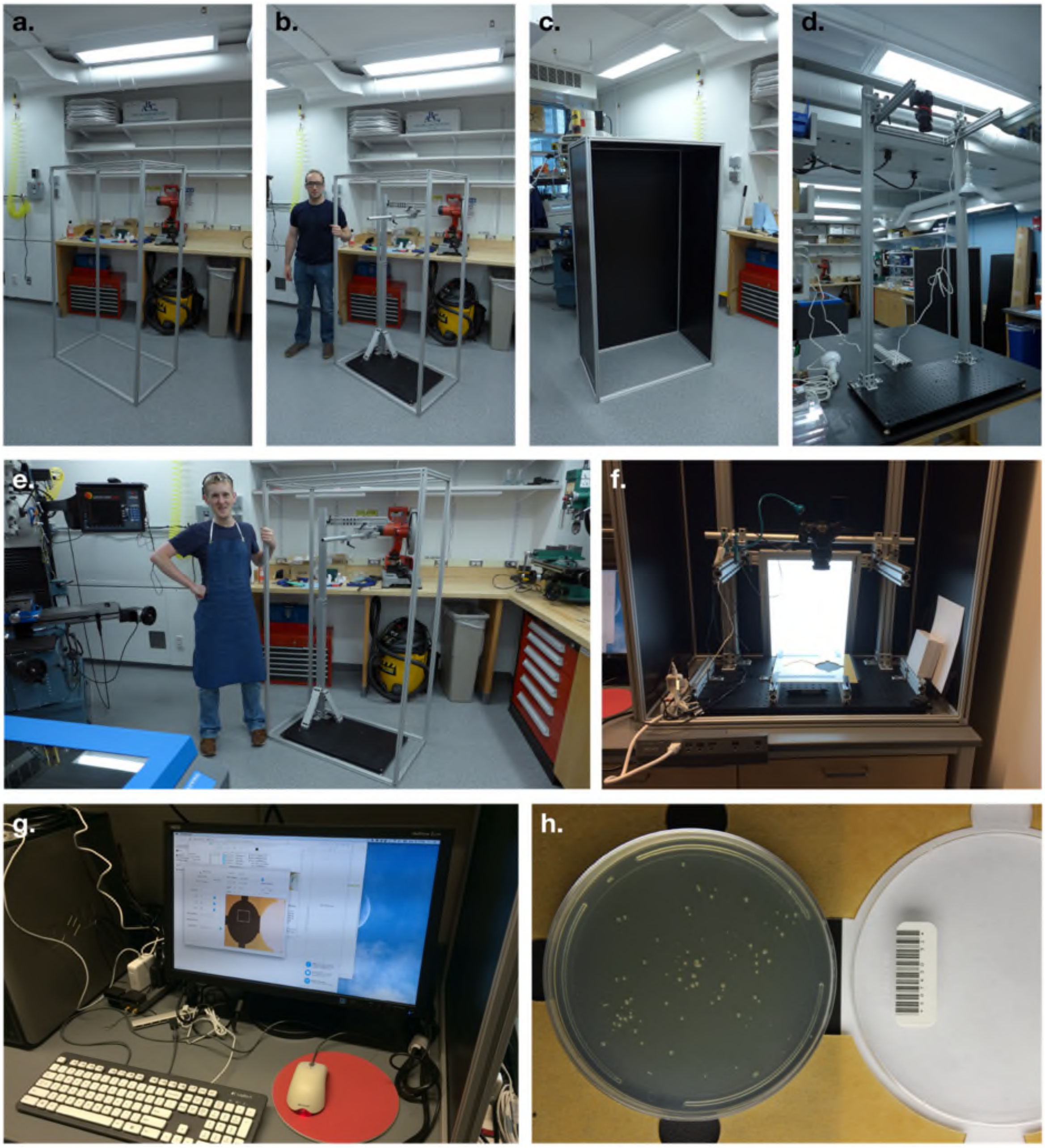

Figure S14. Construction and use of the large format macroscope. The large format macroscope is used to photograph petri dishes and 96-well storage plates where cultures of mutant libraries are initially grown before transfer to assay plates. The height of the macroscope allows the camera to be placed high above the sample, limiting perspective distortion. (**a**) Macroscope enclosure frame made from T-slot. (**b**) Michael Baym next the macroscope enclosure for scale. Baym is ≈ 1.75 m tall (with permission from Michael Baym, and photographer Buz Barstow). (**c**) Enclosure with 0.25" plastic sides fitted. Sides were fabricated by Altec Plastics in Boston, MA. (**d**) Macroscope internal frame. We decided to support the camera with two rails rather than a cantilever to minimize vibration. The base is a 1/4-20 tapped optical breadboard (Newport Corporation). (**e**) Barstow next to macroscope frame (with permission from Buz Barstow, and photographer Michael Baym). (**f**) Assembled macroscope. (**g**) Macroscope data acquisition software running on a Mac Mini computer. (**h**) Petri dish photographed by macroscope.

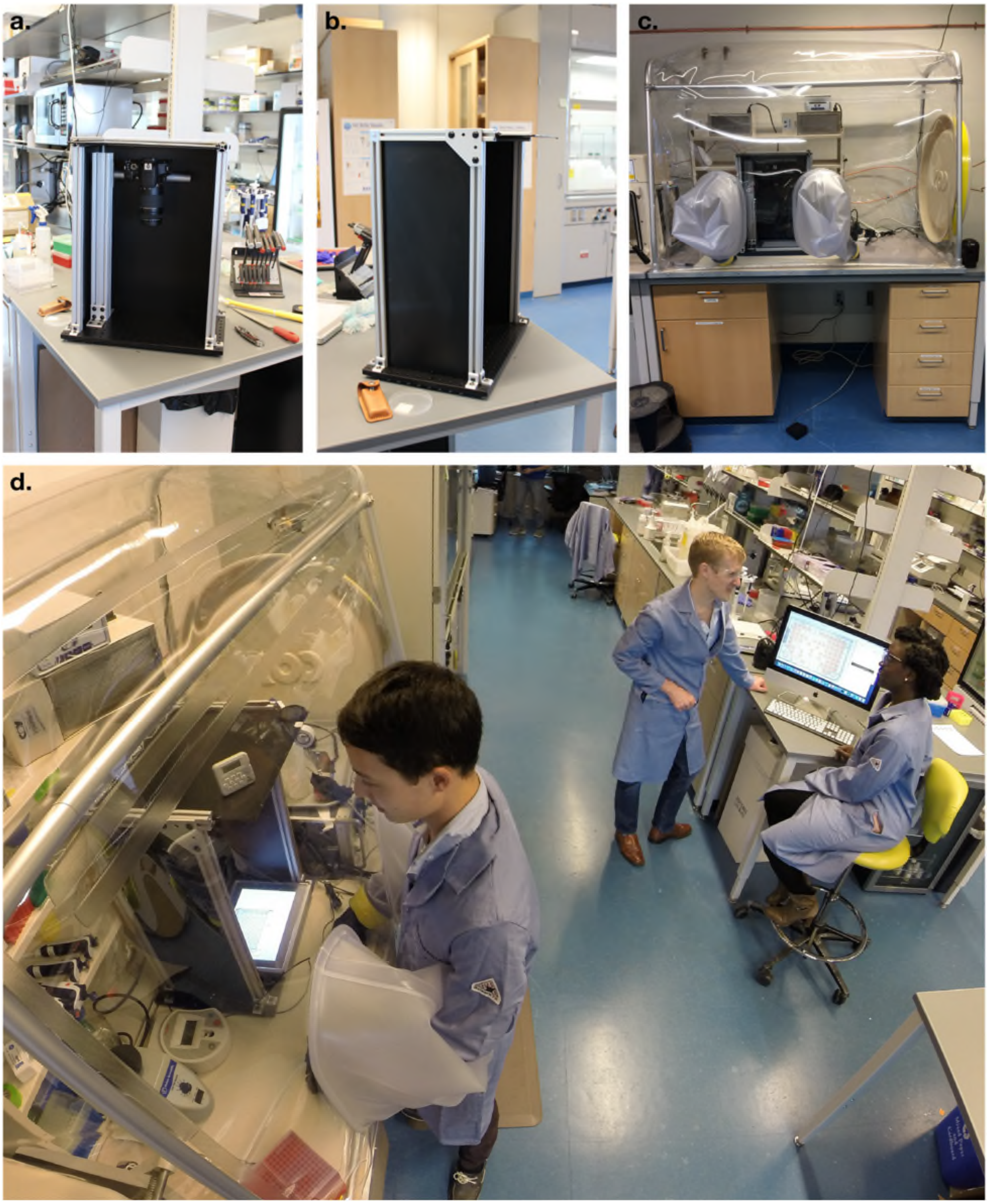

Figure S15. Construction and use of second generation miniature macroscope. (**a** and **b**) The miniature macroscope was built on an optical breadboard and enclosed on four sides. The camera is mounted on a cantilever tube to allow for up-down, left-right and rotational adjustment. (**c**) The macroscope inside a vinyl bag anaerobic chamber. Note the foot pedal (vPedal) that operates the camera shutter. (**d**) Anzai, Adesina and Barstow operate the macroscope (with permission from Adesina, Anzai and Barstow, and from photographer Todd Reichart, Princeton University).

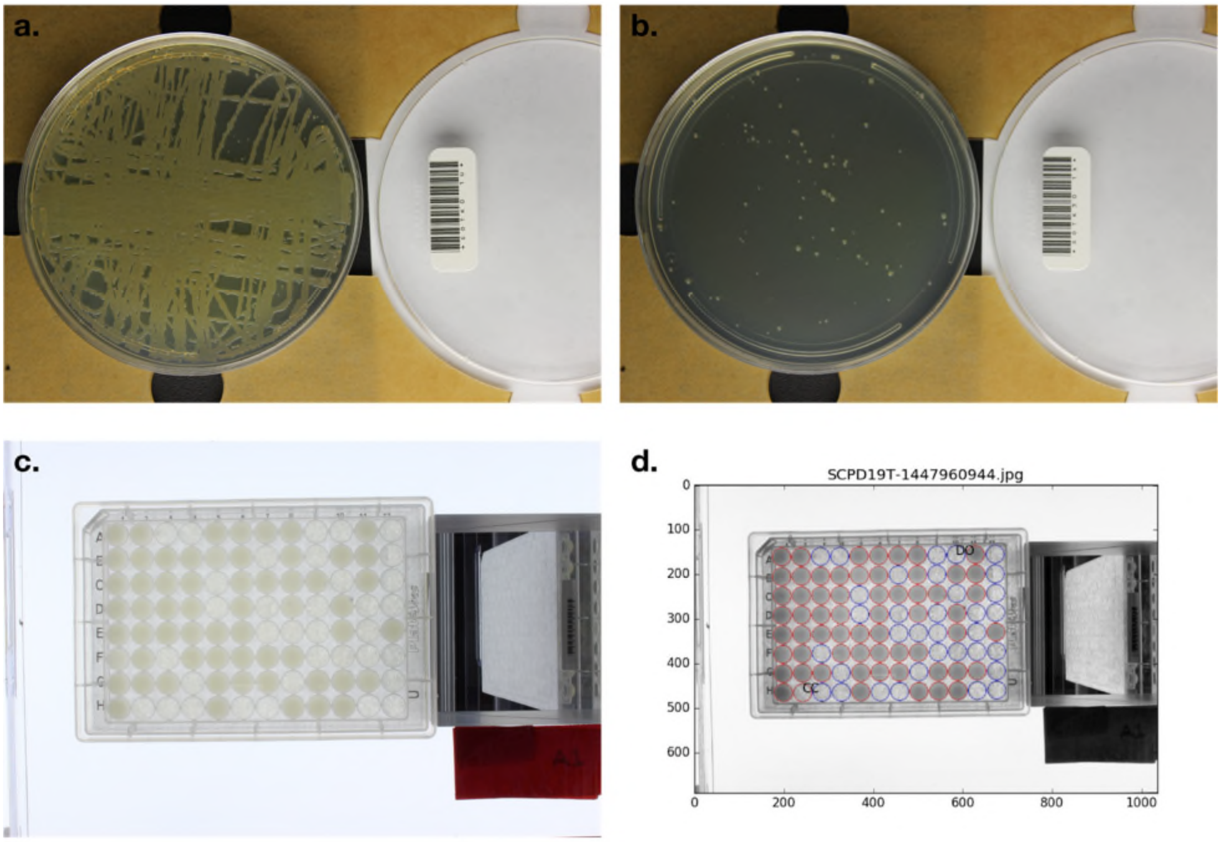

Figure S16. Sample of images from the macroscope, part one. (**a**) *Shewanella oneidensis* grown on LB agar with no kanamycin. (**b**) *S. oneidensis* with 30 µg mL^{-1} kanamycin. Images (**a**) and (**b**) were part of a series that was automatically analyzed to determine minimum kanamycin concentration needed to select for transposon insertion mutants. Note the barcode in the images for sample identification. (**c**) and (**d**) Raw and processed images of a duplicate of *S. oneidensis* knockout collection plate 19 grown on a polypropylene storage plate with an Aeraseal membrane taken from below the plate. An automatic image recognition algorithm was used that checks apparent cell growth in the well (apparently occupied wells are outlined in red, apparently empty wells in blue) with the collection catalog to check for possible cross-contamination (CC) and drop-out (DO) events. For example, a mutant was supposed to have grown in well A10, but did not. There is possibly a small amount of growth in well H2 where there should be none (although mis-identification sometimes happens). This pre-screen of the duplicated collection allows for elimination of a large fraction of false positives, and warns about the possibility of false negatives in the $AHDS_{red}$ oxidation assay. For example, well A10 would appear as a hit (a false positive) in the $AHDS_{red}$ oxidation assay because no oxidation would occur as no cells were transferred to the assay plate. A large number of cross-contamination events (typically 3 or more) could indicate widespread contamination on the plate, which could mask mutants unable to oxidize $AHDS_{red}$ (a false negative). In cases where this happened, we re-duplicated the plate.

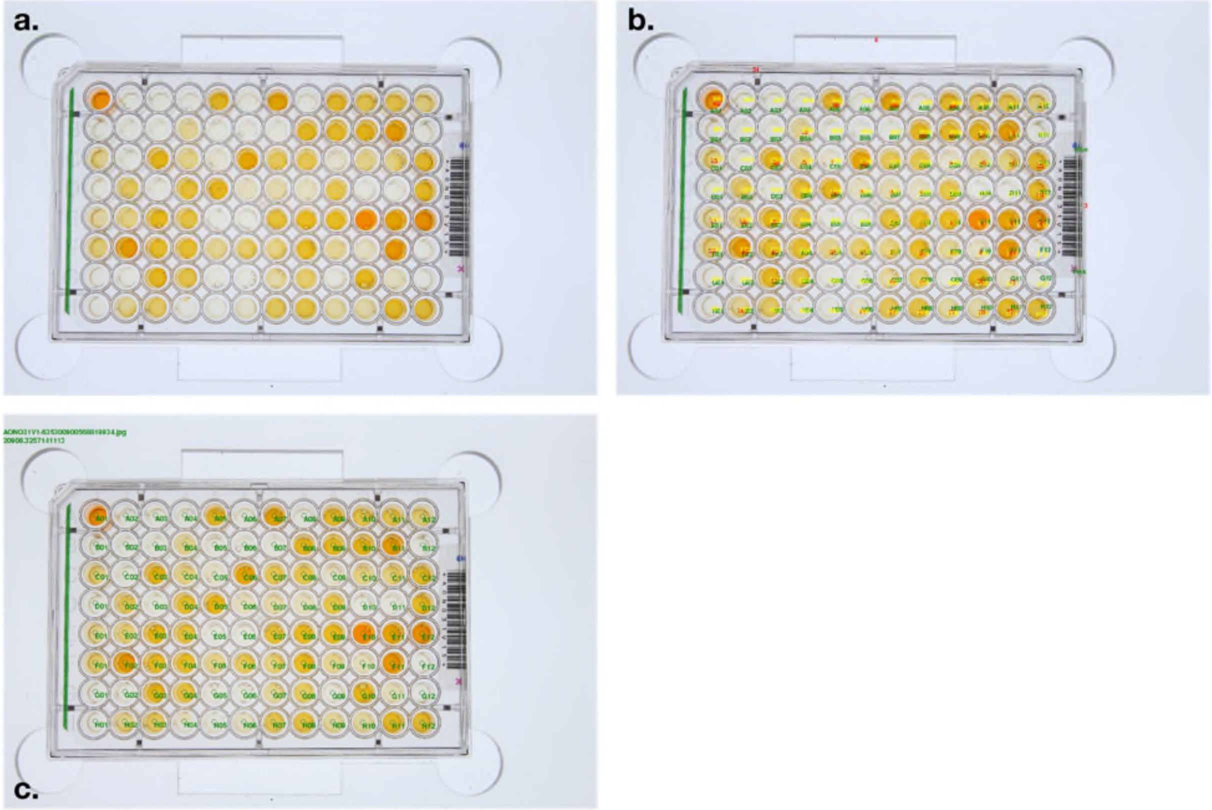

Figure S17. Sample of images from the macroscope, part two. (**a**) Raw image of $AHDS_{red}$/$AQDS_{ox}$ redox assay plate. (**b**) Processed image of assay plate with initial estimates for well identities and positions in green (the well center is to the top left of the text), and refined well positions in yellow. (**c**) Processed image of the assay plate with circles indicating area for color sampling.

Table S1. Electrochemical data observed on cathodes for selected *S. oneidensis* transposon insertion mutants and controls. Averages are calculated from the mean of $n \geq 3$ replicates. Errors are calculated as ±one standard deviation of the mean.

**Strain**	**Average Cathodic Current (µA)**	**Average Biological current (µA)**	**Average Anodic Current (µA)**	**Average Protein (µg/mL)**
Wild-type	-22.2 ± 4.0, *n*=6	-8.8 ± 3.0, *n*=6	2.2 ± 0.2, *n*=5	143 ± 30, *n*=6
δSO_2460	-26.4 ± 9.3, *n*=3	-10.7 ± 5.2, *n*=3	1.7 ± 0.3, *n* =8	94 ± 18, *n*=4
δSO_0401	-23.0 ± 3.9, *n*=3	-7.8 ± 1.5, *n*=3	1.9 ± 0.1, *n* =3	70 ± 39, *n*=4
δSO_A0051	-19.9 ± 3.2, *n*=3	-8.1 ± 2.5, *n*=3	1.5 ± 0.3, *n* =6	90 ± 31, *n*=4
δnapA	-25.5 ± 10.4, *n*=3	-7.3 ± 4.2, *n*=3	1.5 ± 0.4, *n* =3	149 ± 12, *n*=3
δSO_0362	-18.1 ± 3.8, *n*=4	-6.6 ± 2.3, *n*=4	2.0 ± 0.7, *n* =6	113 ± 45, *n*=4
δSO_4765	-20.5 ± 4.2, *n*=3	-5.8 ± 3.1, *n*=3	1.9 ± 0.3, *n* =5	91 ± 36, *n*=6
δSO_0302	-23.9 ± 3.7, *n*=3	-5.5 ± 3.1, *n*=3	1.9 ± 0.2, *n* =4	115 ± 32, *n*=2
δnapG	-18.9 ± 6.9, *n*=5	-6.9 ± 3.1, *n*=5	1.7 ± 0.4, *n* =5	75 ± 39, *n*=6
δSO_2102	-24.3 ± 6.2, *n*=5	-5.7 ± 3.9, *n*=4	1.2 ± 0.4, *n* =5	118 ± 35, *n*=6
δSO_A0110	-10.9 ± 1.7, *n*=4	-4.8 ± 2.4, *n*=4	2.6 ± 1.0, *n*=4	109 ± 75, *n*=5
δSO_4412	-20.4 ± 3.5, *n*=5	-5.5 ± 2.3, *n*=4	1.1 ± 0.6, *n*=5	101 ± 36, *n*=5
δSO_0921	-19.8 ± 1.9, *n*=4	-4.5 ± 0.7, *n*=3	2.2 ± 0.6, *n*=4	96 ± 17, *n*=4
δSO_4766	-13.1 ± 5.4, *n*=4	-4.8 ± 2.6, *n*=4	1.4 ± 0.4, *n*=4	61 ± 18, *n*=5
δSO_0739	-32.3 ± 8.1, *n*=3	-3.8 ± 1.7, *n*=3	2.0 ± 0.6, *n*=5	102 ± 37, *n*=4
δfccA	-21.8 ± 6.7, *n*=6	-4.7 ± 4.7, *n*=6	1.7 ± 0.4, *n*=6	140 ± 62, *n*=6
δcymA	-10.7 ± 0.9, *n*=4	-3.8 ± 1.1, *n*=4	0.4 ± 0.1, *n*=4	70 ± 33, *n*=4
δSO_4149	-21.8 ± 2.5, *n*=3	-3.3 ± 1.0, *n*=3	1.9 ± 0.1, *n*=3	114 ± 27, *n*=3
δSO_4813	-14.3 ± 3.8, *n*=4	-2.8 ± 2.0, *n*=3	1.2 ± 0.3, *n*=7	81 ± 33, *n*=5
δSO_0181	-11.7 ± 3.6, *n*=5	-3.1 ± 1.0, *n*=4	1.7 ± 0.1, *n*=6	128 ± 55, *n*=6
δmtrA	-15.6 ± 0.9, *n*=3	-2.5 ± 0.2, *n*=3	1.1 ± 0.1, *n*=4	99 ± 18, *n*=3
δSO_0841	-17.2 ± 5.1, *n*=6	-2.8 ± 1.8, *n*=4	1.2 ± 0.1, *n*=7	119 ± 34, *n*=6
δSO_0400	-9.2 ± 1.4, *n*=3	-2.3 ± 1.4, *n*=3	1.9 ± 0.3, *n*=3	89 ± 55, *n*=4
δSO_3660	-9.9 ± 0.8, *n*=5	-2.6 ± 0.8, *n*=4	1.7 ± 0.3, *n*=5	81 ± 18, *n*=5
δmtrC	-12.8 ± 2.4, *n*=7	-2.9 ± 1.4, *n*=6	1.2 ± 0.2, *n*=4	120 ± 35, *n*=7

Table S2. Growth and electrochemical data for deletion mutants of *SO_0181*, *SO_0400*, *SO_0841*, *SO_3660*, *SO_3662* and their corresponding complementation strains. Averages are calculated from the mean of *n* ≥ 3 replicates Errors are calculated as ±one standard deviation of the mean. *n.d.* = not detected.

**Strain**	**Aerobic doubling time (± standard deviation)**	**Anaerobic doubling time (± standard deviation)**	**Average Anodic current (µA)**	**Average Cathodic Biological current (µA)**	**Average Midpoint potential (V)**
Wild-type	0.98 ± 0.31	1.41 ± 0.26	2.2 ± 0.2	-8.8 ± 3.0	-0.214 ± 0.01
Wild-type + GFP	0.97 ± 0.24	1.25 ± 0.22	*n.d.*	*n.d.*	*n.d.*
ΔSO_0181	1.27 ± 0.25	1.15 ± 0.24	2.2 ± 0.1	-1.9 ± 1.0	-0.194 ± 0.01
ΔSO_0181 + Complement	1.37 ± 0.21	1.95 ± 0.53	1.1 ± 0.1	-11.6 ± 3.7	-0.195 ± 0.03
ΔSO_0400	1.16 ± 0.44	1.39 ± 0.22	1.4 ± 0.8	-2.45 ± 0.8	-0.205± 0.01
ΔSO_0400 + Complement	1.43 ± 0.17	1.93 ± 0.24	1.2 ± 0.1	-8.2 ± 3.2	-0.201 ± 0.02
ΔSO_0841	1.05 ± 0.44	1.71 ± 0.36	2.7 ± 0.2	-3.4 ± 1.7	-0.216 ± 0.01
ΔSO_0841 + Complement	1.50 ± 0.22	2.02 ± 0.46	0.7 ± 0.1	-8.3 ± 2.4	-0.217 ± 0.01
ΔSO_3660	1.55 ± 0.28	1.93 ± 1.08	1.4 ± 0.7	-2.9 ± 1.2	-0.198 ± 0.03
ΔSO3660 + Complement	1.57 ± 0.34	2.08 ± 0.42	0.7 ± 0.1	-7.4 ± 2.2	-0.217 ± 0.01
ΔSO3662	1.65 ± 0.52	1.88 ± 0.79	1.1 ± 0.1	-2.64 ± 1.5	-0.197 ± 0.01
ΔSO3662 + Complement	1.60 ± 0.51	1.72 ± 0.35	0.7 ± 0.1	-7.5 ± 1.3	-0.206 ± 0.02

Supplementary Bibliography

1 Rowe, A. *et al.* Datasets for Identification of a Pathway for Electron Uptake in Shewanella oneidensis. *Zenodo*, doi:10.5281/zenodo.5013687 (2021).
