# Supplementary material for: Identification of a pathway for electron uptake in Shewanella oneidensis
Source: Commun Biol. 2021 Aug 11;4:957. doi: 10.1038/s42003-021-02454-x (PMC8357807; doi:10.1038/s42003-021-02454-x)
Supplement: Supplementary file 3 — Description of Additional Supplementary Files [file 42003_2021_2454_MOESM3_ESM.pdf]

Description of Additional Supplementary Files

File Name: Supplementary Data 1

Description: A whole genome screen of the *S. oneidensis* whole genome knockout collection found 150 hits that disrupted $AHDS_{red}$ oxidation. The table shows the results of $AHDS_{red}$ oxidation screens with fumarate and nitrate terminal electron acceptors. A 'Y' in column H or I indicates that the mutant was either slow at oxidizing $AHDS_{red}$ or completely failed to do so with fumarate (column H) or nitrate (column I). A 'N' in either column indicates that the rate of $AHDS_{red}$ oxidation was not noticeably different from the wild-type oxidation rate for that terminal electron acceptor.
A 'Y*' in column H or I indicates that slowing or complete failure of $AHDS_{red}$ oxidation was observed but unreliable for that mutant and terminal electron acceptor. Typically, the mutant would only slow or eliminate $AHDS_{red}$ oxidation in ≈ 50% of experimental replicates. Several of these unreliable mutants were selected for electrochemical testing because of an interesting annotation. 'ND 'in either column indicates that data for that combination of mutant and terminal electron acceptor is unavailable.

Disruption Location (column D) indicates the genomic coordinates of the transposon mutant for the locus. In some cases, multiple mutants that caused $AHDS_{red}$ oxidation slowing or failure were identified for a locus. Genomic coordinates between 1 (origin or replication of chromosome) and 4,969,811 refer to nucleotides in the *S. oneidensis* chromosome, and coordinates between 4,969,812 (origin of replication of megaplasmid) and 5,131,424 refer to nucleotides in the *S. oneidensis* megaplasmid. The nominal location column (column E) is used for spreadsheet sorting.

Fractional Distance (column F) indicates the distance of the transposon into the coding region of the locus. It is calculated by finding the distance between the first base of the start codon and the transposon insertion junction and dividing by the length of the gene.

File Name: Supplementary Data 2
Description: Sequence descriptions and metadata for homologs of *SO_0841*, *SO_0181*, *SO_3660*, *SO_3662* and *SO_0400* used for phylogentic analysis.
